# Is treatment adherence linked to self-compassion in schizophrenia?

**DOI:** 10.1192/j.eurpsy.2024.1506

**Published:** 2024-08-27

**Authors:** S. Laabidi, O. Abidi, A. Aissa, R. Hosni, U. Ouali, R. Jomli

**Affiliations:** Psychiatry A, Razi hospital, MANOUBA, Tunisia

## Abstract

**Introduction:**

Self-compassion is defined as the ability to be open to and touched by one’s suffering and to relate to it with kindness and non-judgmental awareness. Although identifying factors related to treatment adherence remains an important challenge in patients with schizophrenia spectrum disorders, self-compassion has rarely been investigated in this population. Further studies are needed to investigate whether self-compassion training can improve treatment adherence in this population.

**Objectives:**

The objective of the present study was to investigate the relationship between self-compassion and treatment adherence in patients with schizophrenia spectrum disorders.

**Methods:**

thirty stabilized adult outpatients with schizophrenia (n=18), schizoaffective disorder (n=11), brief psychotic disorder (n=1) per DSM-5 criteria were included. Self- compassion was assessed using the 26-item Self-Compassion Scale (SCS). Treatment adherence was assessed using the Medical Adherence Rating Scale (MARS). Socio- demographic characteristics, including age, gender, academic level, and mean daily antipsychotic dosages were collected.

**Results:**

There was no significant difference in SCS scores and MARS scores as a function of gender, age, or academic level. The results of the present preliminary study suggest a positive correlation between the SCS total scores and the MARS scores. It was found that higher levels of self-compassion are related with higher levels of treatment adherence in patients with schizophrenia spectrum disorders and lower levels of self- compassion are associated with discontinuation of medications without a psychiatrist’s recommendation. This connection was present in all diagnostic groups.

**Conclusions:**

The results of the present preliminary study suggest that self-compassion and treatment adherence are closely related. Improving self-compassion in patients with schizophrenia spectrum disorders may improve their level of treatment adherence. Further studies are needed to investigate whether self-compassion training programs could be useful as an extension of standard psychoeducation and cognitive behavioral therapy to improve treatment adherence in this population.

**Disclosure of Interest:**

None Declared

